# Computed Tomography in Trauma Patients Accepted in Transfer: Missed Injuries and Rationale for Repeat Imaging. Can we do Better?

**Published:** 2019-02-26

**Authors:** Seth A. Vernon, Stephen D. Helmer, Jeanette G. Ward, James M. Haan

**Affiliations:** 1 Mowery Clinic, Salina, KS; 2 University of Kansas School of Medicine-Wichita, Department of Surgery; 3 Via Christi Hospital Saint Francis, Wichita, KS; 4 Chandler Regional Medical Center, Chandler, AZ

**Keywords:** trauma centers, patient transfer, hospital referrals, computed x-ray tomography

## Abstract

**Introduction:**

Computed tomography scans often are repeated on trauma patient transfers, leading to increased radiation exposure, resource utilization, and costs. This study examined the incidence of repeated computed tomography scans (RCT) in trauma patient transfers before and after software upgrades, physician education, and encouragement to reduce RCT.

**Methods:**

The number of RCTs at an American College of Surgeons Committee on Trauma verified level 1 trauma center was measured. The trauma team was educated and encouraged to use the computed tomography scans received with transfer trauma patients as per study protocol. All available images were reviewed and reasons for a RCT when ordered were recorded and categorized. Impact of system improvements and education on subsequent RCT were evaluated.

**Results:**

A RCT was done on 47.2% (n = 76) of patients throughout the study period. Unacceptable image quality and possible missed diagnoses were the most commonly reported reasons for a RCT. Preventable reasons for a RCT (attending refusal to read outside films, incompatible software, and physician preference) decreased from 25.8 to 14.3% over the study periods.

**Conclusions:**

The volume of unnecessary RCT can be reduced primarily through software updates and physician education, thereby decreasing radiation exposure, patient cost, and inefficiencies in hospital resource usage.

## INTRODUCTION

Regional Trauma Centers (RTCs) accept a large number of trauma patients in transfer, many of whom arrive with imaging studies, in part, due to the increasing number of computed tomography (CT) scanners that have become available throughout rural America.^1^,^2^ What formerly was “stabilize and ship” has morphed into “stabilize, CT scan, and ship.” Justifiable reasons for obtaining CTs at outlying hospitals prior to transfer include the need to identify injuries, properly triage patients, determine the appropriate transport method,^3^ and document the need for referral to a RTC.^4^ Many RTCs have formal or informal protocols to perform a total body CT or pan-scan on all trauma patients accepted in transfer, which often duplicates some or all of the imaging performed pre-transfer. The preconceived notion that rural hospitals produce suboptimal CT images may have contributed to these strategies.

There are multiple interests driving the effort to decrease duplicate imaging.^5^ One reason is a heightened public awareness of the risks associated with CT, primarily the exposure to ionizing radiation and increased risk of malignancy.^6^–^9^ In addition to radiation exposure, intravenous contrast administration often is duplicated. The overall volume of contrast infused is an independent risk factor for contrast-induced nephropathy and should be minimized.^10^,^11^ Uncommon but potentially devastating, non-renal sequelae of intravenous contrast such as allergic reactions and intravenous access site extravasations also can occur.^12^,^13^ Another major factor driving efforts to reduce unnecessary repeat imaging is the awareness of the over-consumption of resources. Many institutions recognize the financial burden of duplicated imaging of trauma patients.^6^–^9^,^14^ In response to the widespread concern regarding repeat imaging, multiple professional societies have responded by publishing recommendations regarding minimizing radiation dose and educating patients prior to imaging.^15^

The purpose of this study was to determine if CT scans from transferring hospitals can be used at the time of patient evaluation, identify any obstacles that impair the effective use of those CT scans, determine physician reasoning behind repeating CT scans, identify any missed injuries or complications associated with using pre-transfer CT scans, and determine if software updates and physician education can reduce RCT effectively for preventable reasons.

## METHODS

As part of a quality improvement initiative, we prospectively collected data on all trauma patients accepted in transfer to our American College of Surgeons Verified Level I Trauma Center during two time periods in 2009 and 2010. During time period 1 (T1: July 1, 2009 to November 30, 2009), the importance of use of outside imaging studies was addressed with the surgery residents and trauma attendings at peer review as a quality initiative. During T1, physicians were aware that they were being observed. During a subsequent five-month wash-out interval, this education was followed by installing imaging software on all trauma bay computers to speed image loading and ease of use. These software upgrades were intended to reduce the number of transferred scans that were unreadable due to hardware or software incompatibility. Finally, hands on training was performed with technologists, residents, and attendings, who then provided training to those that followed. Following the wash-out period of five months, a second time period (T2: March 11, 2010 to May 3, 2010) of follow-up data collection was performed to compare findings from T1. The data from T2 was collected retrospectively, and physicians were unaware that data from T2 were being evaluated in order to assess the effectiveness of software upgrades and additional training and physicians were blinded to study purpose.

The attending trauma surgeon or resident collected quality assurance data at the time of patient arrival in the trauma bay during T1. Each patient was evaluated and the accompanying image(s) reviewed. A data sheet was completed on all patients accepted in transfer who underwent pre-transfer CT imaging. Data variables included patient demographics (age, race, and gender), trauma activation level, which body region(s) were CT imaged prior to arrival, presence of CD containing images (radiology report alone was unacceptable), if the image was read by a resident or attending physician, if a repeat CT was ordered and what type of CT it was, and the reasoning given for the repeated CT.

Reasons for performing another CT are detailed in Table 1. Physicians were allowed to give more than one reason for repeating a scan.

The data collected during T1 were used to generate a quality assurance database to evaluate resource utilization with regard to CT scans. The database was reviewed retrospectively in conjunction with each patient’s electronic health record and trauma registry. We hypothesized there would be an overall decrease, from T1 to T2, in the number of CTs repeated and the pre-transfer CT scans could be used safely during real-time patient evaluation with a low risk of missed injuries.

### Satistical Analysis

Data were analyzed using chi-square analyses on SPSS release 19.0 (IBM Corp., Somers, New York). All statistical tests were two-sided and considered significant when the resultant p value was ≤ 0.05. This study was approved by the Institutional Review Board of Via Christi Hospitals Wichita, Inc. and the Human Subjects Committee of the University of Kansas School of Medicine-Wichita.

## RESULTS

The quality assurance database included 142 patients during T1 and 27 patients during T2 for a total of 169 patients (Table 2). During T1, three patients did not arrive with a CD containing their images, and five patients arrived with a CD that did not include all the documented scans. During T2, one patient did not arrive with a CD containing their images. Therefore, 94.4% (n = 134) and 96.3% (n = 26) of our trauma patients, respective to T1 and T2, arrived in the trauma bay with a CD that contained all of their locally obtained scans.

Of those patients that arrived with pre-transfer CTs, 46.9% (n = 75) went from our trauma bay to radiology to obtain a repeat CT scan. No statistically significant difference was observed in the repeat CT rate between T1 and T2 (46.3 vs 50.0%, p = 0.727); however, the reasons behind repeating scans changed. Table 3 includes a comparison of the reasons given for repeating CT scans between T1 and T2. The most common reasons for repeat CT in T1 were unacceptable image quality (47.0%) and possible missed diagnosis (36.4%). In T2, the most common reasons for repeat imaging were possible missed diagnosis (42.9%), progression of injury (21.4%), and additional studies needed (21.4%). From T1 to T2, there was a significant decrease in repeat imaging for unacceptable image quality (47.0 to 14.3%, p = 0.024) and a concurrent increase in repeat imaging due to progression of injury (3.0 to 21.4%, p = 0.035) and additional studies needed (3.0 to 21.4%, p = 0.035).

Adverse outcomes related to using pre-transfer CT scan were defined as injuries not visualized on the outside CT scan or injuries incorrectly characterized on that imaging, effecting management. After reviewing the trauma registry and medical records, no missed injuries or adverse outcomes related to using pre-transfer CT scans were identified.

## DISCUSSION

The overall rate of patients undergoing repeat CT scans was 46.9%, which was similar to other recently published data of 53 to 58%.^1^,^7^,^14^ Haley et al.^7^ found that 53% of referrals underwent repeat imaging at their trauma center, costing an additional $610,000 on duplicated CT imaging at an average cost of $2,985 per patient. These findings were comparable to those from Cook et al.^8^ in which the additional charge generated from repeating a CT scan of the abdomen was $3,055 per scan. Most recently, Gupta et al.^14^ highlighted this “inefficiency in rural trauma” with regard to repeat CT imaging. They found that 58% of their patients underwent a repeat CT scan with reasons similar to those reported in our study. Our findings are congruent and suggest repeat imaging is prevalent among rural trauma centers and not isolated to any geographic region of the United States.

Surprisingly, 95.3% of our patients arrived with a CD containing all their pre-transfer CT imaging. In similar studies, 12 to 20% of scans were not sent with the patient or not viewable due to software incompatibility.^5^,^14^ Our “incompatible software” category accounted for 10.0% of the overall reasons for repeating a CT and was not significantly different between study time periods. Our observed decrease in “unacceptable” images was likely due to the persistent intervention occurring throughout our study time periods involving increased physician awareness, computer literacy, and software upgrades.

One of the most important issues surrounding pre-transfer CT scans was the potential for missed injuries because rural imaging was purported to be sub-optimal or inadequate. A recent survey indicated that while greater than 90% of rural critical access hospitals have access to CT, the equipment is more likely of lower quality with less resolution, such as 1 to 4 slice scanners.^2^ Despite the disclaimer printed on the CD that “these images should not be used for diagnostic purposes”, our experience was that the overall resolution and quality was acceptable. During T2, 14.3% of the imaging studies fell into the “unacceptable quality” category. Anecdotally, this related more to contrast timing or motion artifacts, which are technologist dependent, as opposed to hardware or software issues. After reviewing patient medical records and the trauma registry, no missed injuries related to using pre-transfer CTs were identified.

When considering the use of repeat CT scans, the risk of missed injuries must be weighed against increased radiation exposure. The increased risk of radiography-induced cancer from CT scans is well documented in the younger population.^16^ Recent computer models indicate middle-aged individuals are susceptible to radiography-induced cancer, and the risk may be twice what was thought previously.^16^,^17^ Decreasing the rate of repeating those particular studies would decrease the patient’s cumulative radiation dose and contrast exposure.^18^ While repeated CTs were done less often for unacceptable images during T2, the proportion of “additional studies needed” increased. Additional studies were included as repeat scans because there was considerable overlap in the scan itself with duplicated radiation and risks. As an example, patients included in this category may have arrived with only a chest CT, but also needed imaging of the abdomen/pelvis. The anatomic overlap of separately imaging the neck, chest, abdomen, and pelvis results in an increased radiation dose to bordering tissues. Due to both observed decreases in some reason categories, such as “unacceptable quality,” and concurrent increases in others, such as “additional studies needed,” there was no overall decrease in RCT in the current study. We deem our findings still to indicate success as we observed decreased RCT for preventable reasons while seeing a concurrent increase in nonpreventable reasons that may have multiple explanations.

Our current practice has evolved based on the results of this study. For patients arriving in transfer with a CD, the images are viewed immediately on the trauma bay computer, then given to the radiology technologist to upload into our Picture Archiving and Communication System (PACS). Within 30 minutes, the images are viewable through our local software on any hospital computer. Our radiologists do not over-read outside CT scans routinely unless a clinical scenario prompts a special request from the trauma team. However, the images are archived and follow-up images are compared to the pre-transfer imaging, which is often the case for follow-up head CTs.

Teleradiology, which allows digital transfer of images from the referral hospital for review by the receiving in-house trauma team, will influence interfacility transfer and management of acutely injured patients.^3^ One study showed that an integrated trauma system that utilizes PACS has significantly fewer repeat CT scans (16%) when compared to a non-integrated system (48%).^19^ Because the integrated trauma center received a third of patients from hospitals outside the integrated trauma system, the authors suggested that the 16% of scans repeated at the integrated hospital may be much lower for populations transferring from hospitals within the integrated trauma system.

Several limitations existed within this study. First, the findings came from a single RTC within a small time period. Second, we did not know which, or how many, pre-transfer CT scans were indicated based on Advanced Trauma Life Support or current trauma practices. Perhaps some scans were not repeated because they were not indicated to begin with. However, our findings were comparable to those previously reported at other institutions from various geographic regions.^5^,^7^,^14^ We did not measure or quantify radiation exposure or IV contrast exposure nor their associated complications. We also defined “repeat” as any trip to the CT scanner from our trauma bay after the patient was scanned at the transferring facility previously. The “progression of injury” category contained five patients and could be excluded, because the reason for their trip to the scanner is independent of pre-transfer imaging. Additionally, the number of observations in some categories evaluated, as well as in T2, were low enough that it may limit their generalizability.

Future work on this topic revolves around educating our transferring hospitals with regard to CT imaging protocols. The idea that small rural hospitals do not have adequate technology to produce quality CT scans is not supported by our findings. It seems unreasonable to suggest that rural hospitals not perform CT scans on trauma patients. Rather, they should be educated with regard to current imaging protocols, and our trauma systems should be refined to incorporate teleradiology programs that will aid triage and transfer.

## CONCLUSIONS

With appropriate software and practitioner effort pre-transfer, the majority of CTs may be used effectively and safely at the time of patient presentation to regional trauma centers and need for RCT for preventable reasons can be minimized.

## Figures and Tables

**Table 1:** Reasons for performing a repeat CT.

Poor quality/unacceptable images, including CTs with poorly timed contrast, non-contrasted scans of the chest/abdomen/pelvis, no neck reconstructions, or blurry images from excessive motion artifact.
Possible missed injury, including patients with cervical spine fractures that needed a CT angiogram of the neck or those patients with pelvic fractures, lower rib fractures or spine fractures that needed CT imaging of their abdomen/pelvis.
Incompatible software, including images that could not be loaded, windowed, scrolled, or viewed satisfactorily due to software issues.
Additional studies were needed for patients who had incomplete imaging, including CTs of the upper abdomen that did not include the pelvis or a patient with an adequate CT chest, but also needed an abdomen/pelvis scan.
Progression of injury, including patients who arrived with a worse clinical picture, inconsistent with their imaging.
Physician preference/other served as a miscellaneous category to repeat a scan for an unclassified reason.

**Table 2:** Comparison of computed tomography (CT) scans received in transfer, proportion receiving repeat CT and ordering patterns of repeat CTs for patients injured in time periods 1 and 2.

Parameter	Period I Number (%)	Period II Number (%)	p value
Number of observations	142 (84.0)	27 (16.0)	
Status of CT’s			0.548
All arrived with patient	134 (94.4)	26 (96.3)	
Some, but not all, arrived with patient	5 (3.5)	0 (0.0)	
None arrived with patient	3 (2.1)	1 (3.7)	
Patient CT’s repeated	62/134 (46.3)	13/26 (50.0)	0.727
CT reordered by	N = 61	N = 13	0.824
Resident	40 (65.6)	9 (69.2)	
Attending surgeon	17 (27.9)	4 (30.8)	
Radiologist	3 (4.9)	0 (0.0)	
Neurosurgeon	1 (1.6)	0 (0.0)	

**Table 3:** Comparison of reasons for repeat computed tomography (CT) scans for patients injured in time periods 1 and 2.

Parameter	Period I Number (%)	Period II Number (%)	p value
Number of observations*	N = 66	N = 14	
Reason for repeat CT			
Unacceptable quality	31 (47.0)	2 (14.3)	0.024
Possible missed diagnosis	24 (36.4)	6 (42.9)	0.649
Attending refused to read outside film	10 (15.2)	0 (0.0)	0.196
Incompatible software	6 (9.1)	2 (14.3)	0.624
Additional studies needed	2 (3.0)	3 (21.4)	0.035
Progression of injury	2 (3.0)	3 (21.4)	0.035
Physician preference	1 (1.5)	0 (0.0)	1.000
Patient condition	1 (1.5)	1 (7.1)	0.321
Radiologist refused to read outside film	0	0	----

* Multiple reasons for repeating a CT image or set of images sometimes were given for a single patient.
